# Reverse transcription of the pFOXC mitochondrial retroplasmids of *Fusarium oxysporum *is protein primed

**DOI:** 10.1186/1759-8753-2-1

**Published:** 2011-01-21

**Authors:** Jeffrey T Galligan, Sarah E Marchetti, John C Kennell

**Affiliations:** 1 Department of Biology, Saint Louis University, St Louis, MO, USA; 2 Department of Pathology, Harvard Medical School, Boston, MA, USA; 3 Department of Biology, Washington University in St Louis, St Louis, MO, USA

## Abstract

**Background:**

The pFOXC retroplasmids are small, autonomously replicating DNA molecules found in mitochondria of certain strains of the filamentous fungus *Fusarium oxysporum *and are among the first linear genetic elements shown to replicate via reverse transcription. The plasmids have a unique clothespin structure that includes a 5'-linked protein and telomere-like terminal repeats, with pFOXC2 and pFOXC3 having iterative copies of a 5 bp sequence. The plasmids contain a single large open reading frame (ORF) encoding an active reverse transcriptase (RT). The pFOXC-RT is associated with the plasmid transcript in a ribonucleoprotein (RNP) complex and can synthesize full-length (-) strand cDNA products. In reactions containing partially purified RT preparations with exogenous RNAs, the pFOXC3-RT has been shown to initiate cDNA synthesis by use of snapped-back RNAs, as well as loosely associated DNA primers.

**Results:**

The complete sequence of the distantly related pFOXC1 plasmid was determined and found to terminate in 3-5 copies of a 3 bp sequence. Unexpectedly, the majority of (-) strand cDNA molecules produced from endogenous pFOXC1 transcripts were attached to protein. *In vitro *experiments using partially purified pFOXC3-RT preparations having a single radiolabeled deoxyribonucleotide triphosphate (dNTP) generated a nucleotide-labeled protein that migrated at the size of the pFOXC-RT. The nucleotide preference of deoxynucleotidylation differed between pFOXC3 and pFOXC1 and showed complementarity to the respective 3' terminal repeats. In reactions that include exogenous RNA templates corresponding to the 3' end of pFOXC1, a protein-linked cDNA product was generated following deoxynucleotidylation, suggesting that reverse transcription initiates with a protein primer.

**Conclusions:**

The finding that reverse transcription is protein primed suggests the pFOXC retroplasmids may have an evolutionary relationship with hepadnaviruses, the only other retroelement family known to initiate reverse transcription via a protein primer. Moreover, the similarity to protein-primed linear DNA elements supports models in which the terminal repeats are generated and maintained by a DNA slideback mechanism. The ability of the pFOXC-RT to utilize RNA, DNA and protein primers is unique among polymerases and suggests that the pFOXC plasmids may be evolutionary precursors of a broad range of retroelements, including hepadnaviruses, non-long terminal repeat (non-LTR) retrotransposons and telomerase.

## Background

Retroplasmids are autonomously replicating genetic elements that represent a lineage of mobile elements that replicate via reverse transcription. Thus far, retroplasmids have only been found in mitochondria of filamentous fungi and, like mitochondrial DNA plasmids, they exist in both linear and circular forms (reviewed in [1]). As a group, retroplasmids are relatively small and simple. They range in size from 1.9 to approximately 4.0 kb, and have a single open reading frame (ORF) that encodes a reverse transcriptase (RT). Retroplasmid RTs lack domains that are often associated with other RTs, such as an RNAse H domain, and are thought to be ancestral to a broad range of retroelements as they are deeply rooted within the RT phylogenies [2,3]. Their primitive nature is supported by the mechanisms used to initiate cDNA synthesis; the RT encoded by the circular Mauriceville plasmid of *Neurospora crassa *has been shown to initiate cDNA synthesis *de novo *(that is, without a primer), suggesting that it is mechanistically related to RNA-dependent RNA polymerases [4], whereas the RT encoded by the linear pFOXC3 plasmid of *Fusarium oxysporum *can initiate reverse transcription using RNA or DNA primers having minimal base pairing interactions with templates [5]. The structural and mechanistic features of the plasmids, together with their possible evolutionary origin in the precursors of mitochondria, has led to speculation that mitochondrial retroplasmids represent a type of 'molecular fossil' which has been defined as a contemporary genetic element that is ancient in origin and can reveal information about the evolutionary past [4,6,7].

**Figure 1:**
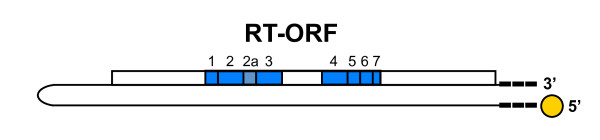
**Schematic diagram of the pFOXC plasmids**. The pFOXC retroplasmids are approximately 1.9 kb linear, double-stranded DNA molecules that have a clothespin structure. They possess a covalently closed hairpin and iterative terminal repeats (black boxes) with a 5'-linked protein (circle). The plasmids have a single open reading frame (ORF) encoding a reverse transcriptase (RT; open box). The location of conserved domains characteristic of reverse transcriptases is indicated by shaded regions 1, 2, 2a, 3-7, with domain 2a also being conserved among non-long terminal repeat (non-LTR) retrotransposons. Details concerning the plasmid ORFs and iterative repeats are given in Table 1.

The pFOXC retroplasmids are 1.9 kb linear DNA molecules found in mitochondria of certain *forma speciales *of the fungal plant pathogen *F. oxysporum*. Plasmids that have been identified to date fall into two homology groups, the pFOXC2/pFOXC3 group and the pFOXC1 group [1,8,9]. The RTs encoded by pFOXC2 and pFOXC3 have 93% amino acid sequence identity within the highly conserved RT domains yet they each share less than 40% sequence identity within the conserved domains of the pFOXC1-RT [6], indicating a relatively high degree of evolutionary divergence between the two homology groups. The plasmid DNAs have a 'clothespin' structure that includes a hairpin at one terminus and telomere-like repeats at the other terminus (Figure 1), with plasmids pFOXC2 and pFOXC3 each having 3-5 copies of a 5 bp sequence (5'-ATCTA; Table 1; [6]). Characterization of *in vivo *replication intermediates showed that the pentameric repeats are transcribed and that reverse transcription begins at or near the 3' end of the plasmid transcript. The number of repeats in (-) strand cDNA intermediates was found to be slightly greater than the number in the plasmid transcripts, suggesting that the maintenance and generation of the terminal repeats occurs during the reverse transcription step of plasmid replication [5]. The association of linear genetic elements having telomere-like repeats that replicate via reverse transcription suggests that the pFOXC plasmids may be contemporary descendants of primitive chromosomes and/or have a direct evolutionary relationship to telomerase. In addition, the 3' repeats of the pFOXC plasmids bear a striking resemblance to 3' tails of certain long and short interspersed elements (LINEs and SINEs, respectively) that have been shown to generate additional repeats during retrotransposition [10,11].

**Table 1:** Features of the pFOXC mitochondrial retroplasmids

Plasmid	**Length**^**a**^	Accession number	ORF size	**Identity to C3-RT (%)**^**b**^	**Terminal repeat**^**c**^
pFOXC1	1,867	HQ026775	497	39	...CAA CAA CAA CAA-3'

pFOXC2	1,905	AF124843	527	93	...ATCTA ATCTA ATCTA AT-3'

pFOXC3	1,836	AF124844	527	100	...ATCTA ATCTA ATCTA AT-3'

^a^Length is an estimate due to the imprecise designation of the 5'-most site and heterogeneity of the 3' end.

^b^Amino acid identity within the region encompassing the highly conserved RT domains 1-7.

^c^Indicates the average number of repeat sequences recovered among 15 or more analyzed clones.

ORF = open reading frame; RT = reverse transcriptase.

The pFOXC plasmids also have a 5'-linked protein, which is reminiscent of a variety of linear DNA and RNA genetic elements that replicate by use of a protein primer. Protein-primed replication has been most thoroughly studied with linear DNA viruses, such as adenovirus and bacteriophage Ф-29 (reviewed in [12,13]). These linear DNA molecules possess a covalently linked 5' terminal protein (TP). During initiation of replication, the TP associates with the viral DNA polymerase and the TP-polymerase complex binds to the genome terminus that includes one or more iterations of a 1-3 nucleotide sequence. The polymerase catalyzes a phosphodiester bond between the initiating nucleotide and the hydroxyl group of a serine, threonine or tyrosine residue of the TP [13]. Linear mitochondrial DNA plasmids also possess covalently bound 5' terminal proteins and replication appears to initiate via protein priming associated with the plasmid encoded DNA polymerase [14,15]. To date, hepadnaviruses are the only retroelement family shown to employ a protein primer. The partially double-stranded, circular hepadnaviral genome encodes an RT that initiates reverse transcription at a specific site of a structured RNA (ε) by use of a tyrosine residue located in the TP domain of the RT itself [16,17]. Similar protein-priming mechanisms have also been reported with certain RNA viruses (for example, polio [18]).

Previous studies using isolated mitochondrial ribonucleoprotein (mtRNP) particles from pFOXC plasmid-containing strains demonstrated that reverse transcription begins opposite the 3' end of the plasmid RNA and can generate full length (-) strand cDNA products [6]. Studies using an *in vitro *system to investigate reverse transcription showed that the pFOXC-RT can initiate cDNA synthesis by the use of snapped-back RNAs or DNA primers. The RT showed preference for DNA primers that bound to the 3'-most terminal repeat of *in vitro *synthesized RNA templates. The RT was also found to copy DNA templates and was able to extend weakly associated primers that had up to three 3' mismatches with the template [5].

Here, we provide evidence that the pFOXC retroplasmids can use a protein primer to initiate reverse transcription. Analysis of reverse transcription reactions using pFOXC1-containing mtRNPs demonstrated that a large portion of the products are associated with protein, and an *in vitro *system using partially purified pFOXC1-RT preparations with exogenous RNA templates corresponding to the 3' end of the pFOXC1 plasmid produced a protein-linked cDNA. Analogous reactions using pFOXC3-containing mtRNPs revealed a nucleotide-linked protein that migrated at the size of the plasmid RT, suggesting that the RT is serving as primer. These findings suggest that the pFOXC plasmids have an evolutionary relationship with protein-primed genetic elements, including hepadnaviruses, a finding that may shed new light on the evolutionary origins of the telomerase complex.

## Results

Prior studies of reverse transcription reactions using mtRNPs from plasmid-containing strains failed to provide evidence that large RNA or DNA molecules are associated with nascent cDNA replication intermediates [6], yet studies of *in vitro *reactions using exogenous templates indicated that the pFOXC3-RT could use snapped-back RNAs or loosely associated DNAs to prime cDNA synthesis [5]. To investigate whether snapped-back RNAs (or possibly small DNAs) are used as a method of initiation *in vivo*, plasmid replication products were re-examined by primer extension analysis in hopes of capturing remnants of nucleic acid primers. For this analysis, total nucleic acids were isolated from mitochondria and used in reactions having an end-labeled primer that is complementary to a region approximately 100 nucleotides downstream of the 5' end of (-) strand cDNAs. To extend the primers, Moloney murine leukemia virus (MMLV)-RT was used as it is capable of copying both RNA and DNA templates, and products were separated on a denaturing polyacrylamide gel. A minor fraction (<5%) of the labeled products extended beyond the site corresponding to the 3' end of the plasmid DNA (data not shown). These products were isolated, amplified by anchored PCR, cloned and sequenced. The sequence of several clones indicated that the primer extension products terminated at the site corresponding to the 3' end of the plasmid and had no additional sequences that might be suggestive of an attached primer. It is likely that a fraction of the primer extension products reassociated with DNA or cDNA templates during electrophoresis or were otherwise held up during the migration into the polyacrylamide gel. These findings, coupled with previous analysis of plasmid replication intermediates [5], failed to provide evidence of nucleic acid primers associated with nascent *in vivo *(-) strand cDNAs.

### Analysis of the distantly related pFOXC1

To gain additional insight into the mechanism of reverse transcription of the pFOXC retroplasmids, experiments were carried out with pFOXC1, which is found in the mitochondria of *F. oxysporum *f. sp. *conglutinans*. Previous reports indicated that the sequence of a 785 bp internal *Bgl*II restriction fragment of pFOXC1 contained a potential open reading frame that has similarity to the RTs of pFOXC2 and pFOXC3 [6,19] yet, the percentage amino acid sequence identity within the highly conserved RT regions was surprisingly low (<40%), indicating that pFOXC1 is distantly related. To complete the sequence of pFOXC1, the terminal regions of the plasmid DNA were cloned using an anchored PCR approach and the multiple clones were sequenced. The complete plasmid DNA was found to be 1,867 bp, which is an approximation as the 3' end of the plasmid DNA is heterogeneous in length (GenBank accession no.: HQ026775; Table 1). The plasmid contains a single, large open reading frame that is predicted to encode a polypeptide of 497 amino acids that has highly conserved domains associated with known reverse transcriptases. The predicted pFOXC1-RT is 30 amino acids shorter than the RTs of pFOXC2 and pFOXC3 and is amongst the shortest functional RTs described to date. Similar to the other retroplasmids, the 3' end of the pFOXC1 plasmid has short repeats but, interestingly, they differ in both length and sequence from the terminal repeats of pFOXC2 and pFOXC3. The sequence of clones of the terminus downstream of the ORF showed that the end terminates with three to five copies of the three base sequence 5' CAA (Table 1 and Additional file 1).

Reverse transcription reactions using mitochondrial ribonucleoprotein (mtRNP) particles isolated from a pFOXC1-containing strain (777) were subjected to a variety of pretreatments and post-treatments as previously described for pFOXC2-containing and pFOXC3-containing strains [6]. Mitochondrial RNPs isolated from the pFOXC1-containing strain had reverse transcriptase activity, as measured by the ability to incorporate [^32^P]-labeled dNTPs into high molecular weight products. The level of activity was comparable to that previously reported for pFOXC2-containing and pFOXC3-containing strains and reverse transcriptase activity was distinguished from DNA polymerase activity by its sensitivity to pretreatment with RNase A and insensitivity to the DNA-dependent DNA polymerase inhibitor actinomycin D (Additional file 2).

**Figure 2:**
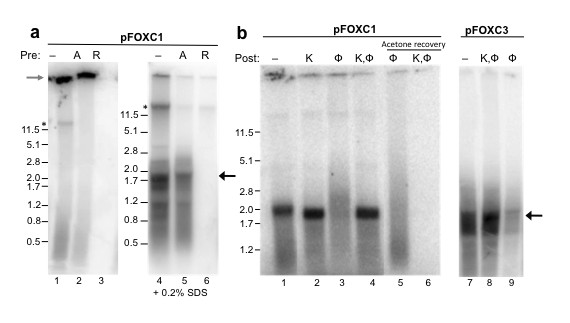
**A portion of pFOXC1 and pFOXC3 endogenous reverse transcription products is associated with protein**. **(a) **Mitochondrial ribonucleoproteins (mtRNPs) from pFOXC1-containing strains were untreated (lanes 1 and 4), or pretreated with actinomycin D (A; lanes 2 and 5) or RNAse A (R; lanes 3 and 6) for 5 min prior to reverse transcription reactions. Following precipitation, products were heated and electrophoresed in 1.2% agarose gels without (lanes 1-3) or with (lanes 4-6) 0.2% SDS in the gel and loading buffer. **(b) **Products from endogenous reverse transcription reactions pretreated with actinomycin D and using pFOXC1-containing (lanes 1-6) and pFOXC3-containing (lanes 7-9) mtRNPs were subjected to the following treatments: lane 1, no treatment; lane 2, incubation with proteinase K (K); lane 3, extraction with phenol-CIA (φ); lane 4, incubation with proteinase K followed by extraction with phenol-CIA (K, φ). Lanes 5 and 6 contain acetone-precipitated products recovered from the organic phase of the phenol extractions shown in lanes 3 and 4, respectively. Minus-strand cDNA products from pFOXC3-containing mtRNPs were subjected to the following treatments: lane 7, no treatment; lane 8, incubation with proteinase K followed by extraction with phenol-CIA (K, φ); lane 9, extraction with phenol-CIA (φ). Products were heated at 65°C in 0.2% SDS followed by electrophoresis in a 1.2% agarose gel containing 0.2% SDS. Marker sizes from 5'-end labeled λ-*Pst*I restriction fragments are indicated in kb pairs on the left. The location of the wells is indicated with a gray arrow. The full-length (-) strand cDNA product is indicated on the right with a black arrow and a high molecular weight band detected in reactions lacking actinomycin D is indicated with an asterisk.

When labeled cDNA products were analyzed on agarose gels, most of the reaction products were unexpectedly retained in the loading well (Figure 2a, lanes 1-3). To prevent protein aggregation, SDS was included in the gel (to a concentration of 0.2%), and reactions were heated in the presence of 0.2% SDS (65°C, 5 min) prior to electrophoresis. This treatment enabled the majority of the labeled products to migrate into agarose gels (Figure 2a, lanes 4-6). The cDNA products were in the size range of 0.5 to 2.0 kb, and a larger product (>12 kb) was also found in reactions that were not pretreated with actinomycin D (Figure 2a, lane 4), which most likely reflects mitochondrial DNA polymerase activity. As expected, labeled products were not generated when the mtRNPs were pretreated with RNase A (Figure 2a, lane 6).

When products of the endogenous reactions were further separated by extending the time of electrophoresis, a major cDNA product was observed that migrated at 1.9 kb, which matches the length of the plasmid RNA. This is analogous to the major products obtained with pFOXC2-containing and pFOXC3-containing mtRNPs that were previously shown to represent (-) strand cDNA products (Figure 2b; [6]). When products of the reverse transcription reactions using pFOXC1-containing mtRNPs were extracted with phenol-chloroform-isoamyl alcohol (CIA), only a small portion were recovered by ethanol precipitation of the aqueous phase and the majority of the cDNA products remained in the organic phase (Figure 2b, lane 3). Treatment of the reaction products with proteinase K resulted in a slight increase in migration compared to untreated reactions (Figure 2b, compare lanes 1 and 2) and prevented cDNA products from being extracted by phenol-CIA (Figure 2b, lane 4). To confirm that the untreated cDNA products extracted with phenol-CIA were trapped in the organic phase, the phenol-CIA layer was precipitated with acetone and labeled products were recovered (Figure 2b, lane 5). In contrast, no labeled products were recovered in the organic phase of extractions from reactions that were post-treated with proteinase K (Figure 2b, lane 6). These results suggest that the products of reverse transcription are attached to protein.

Given the effect that SDS had on the pFOXC1 reaction products, parallel reactions were conducted using mtRNPs containing pFOXC3 (Figure 2b, lanes 7-9). A fraction of cDNAs was removed by extraction with phenol-CIA (Figure 2b, lane 9), suggesting that a smaller, yet significant, portion of pFOXC3 cDNA products is associated with protein and was previously overlooked. Taken together, these results indicate that varying portions of the labeled (-) strand cDNA products derived from endogenous reverse transcription reactions are attached to protein.

### Evidence of protein-primed reverse transcription

Due to the observation that a portion of endogenous (-) strand cDNA products are attached to protein, coupled with the previous demonstration that the plasmid DNA is retained in the organic phase following extraction with phenol, unless pretreated with protease K, and is insensitive to digestion with λ exonuclease (a 5' exonuclease; [6,9]), we hypothesized that the pFOXC-RT uses a protein to prime reverse transcription. To test this hypothesis, reverse transcription reactions were performed using exogenous RNAs that correspond to the 3' end of the plasmid transcripts (that is, 'exogenous' reactions). As described in a previous study [5], endogenous nucleic acids associated with mtRNP particles are first degraded by treatment with micrococcal nuclease (MN) to liberate the plasmid RT. Following chelation of free Ca^++ ^with ethyleneglycol tetra-acetic acid (EGTA), MN-treated mtRNPs are used in reactions having *in vitro *synthesized RNAs together with a [α-^32^P]-labeled nucleotide and a full complement of unlabeled nucleotides. However, in contrast to previous studies in which the labeled products were precipitated with ethanol and resolved on denaturing urea polyacrlyamide gels, exogenous reactions were instead terminated by boiling in Laemmli buffer and products were resolved via SDS-PAGE to examine possible protein-linked cDNA products.

**Figure 3:**
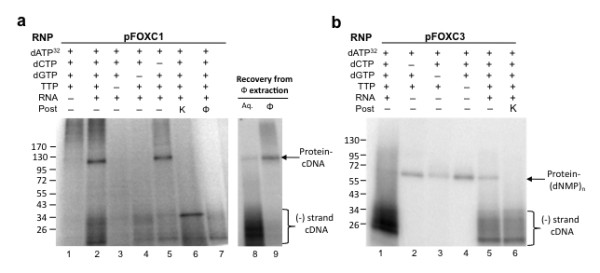
**Protein-primed reverse transcription and identification of a nucleotide-linked protein in exogenous reverse transcription reactions**. Mitochondrial ribonucleoprotein (mtRNP) particles were treated with micrococcal nuclease (MN), incubated with actinomycin D, and used in reactions having [α-^32^P]dATP with various combinations of deoxyribonucleotide triphosphates (dNTPs), either in the absence or presence of an exogenous RNA, as indicated. **(a) **MN-treated pFOXC1-containing mtRNP particles were incubated in the absence (lane 1) or presence of a 92 nucleotide RNA corresponding to the 3' terminus of the pFOXC1 transcript with a full complement of nucleotides (lane 2). Reactions in lanes 3-5 were performed as in lane 2, but a single unlabeled nucleotide was omitted, as indicated. Products from exogenous reactions having a full complement of nucleotides and exogenous RNA were post-treated with proteinase K (K; lane 6), or extracted with phenol-CIA (φ; lane 7), prior to precipitation with ethanol. Products from a duplicate reaction of that in lane 2 were recovered from the aqueous (lane 8) and organic (lane 9) phase. **(b) **MN-treated pFOXC3-containing mtRNPs incubated in the absence (lanes 2-4) or presence (lanes 1, 5 and 6) of a 98 nucleotide RNA corresponding to the 3' terminus of the pFOXC3 transcript with [α-^32^P]dATP and combinations of unlabeled nucleotides, as indicated. Products from a duplicate reaction of that shown in lane 5 were post-treated with proteinase K (K; lane 6). All reactions were boiled in Laemmli buffer prior to separation via 4-20% gradient SDS-PAGE. Prestained protein size markers are indicated on the left in kDa. Protein-linked products and (-) strand cDNA products are indicated on the right.

In reactions using MN-treated pFOXC1-containing mtRNPs, cDNA products were not detected in the absence of an exogenous RNA (Figure 3a, lane 1), yet when a 92 nucleotide transcript that corresponds to the 3' end of the pFOXC1 RNA having 4 copies of the 5' CAA repeat was included, a discrete band was observed that migrated at approximately 120 kDa, as well as non-discrete products migrating below 35 kDa (Figure 3a, lane 2). The omission of thymidine triphosphate (TTP) or deoxyguanosine triphosphate (dGTP) from the reactions prevented the synthesis of both the approximately 120 kDa product and the majority of the products under 35 kDa, whereas the omission of deoxycytidine triphosphate (dCTP) only slightly affected the intensity of the 120 kDa band. Significantly, when the products of a reaction having an exogenous RNA and a full complement of nucleotides were post-treated with proteinase K, the 120 kDa product was eliminated and a prominent band was observed at approximately 34 kDa. When a duplicate reverse transcription reaction was extracted with phenol-CIA, labeled products migrating above approximately 35 kDa were eliminated (Figure 3a, lane 7). To demonstrate that the 120 kDa product was retained in the organic phase following extraction, the phenol-CIA layer was precipitated with acetone and products were resolved on SDS polyacrylamide gels. The 120 kDa product was recovered from the organic phase (Figure 3a, lane 9), which strongly suggests that the labeled product is covalently attached to protein. In contrast, the non-discrete reverse transcription products that migrate below 35 kDa were neither affected by proteinase K treatment nor removed by extraction with phenol-CIA, indicating that they are not associated with a protein and are likely cDNAs that were initiated from snapped-back RNAs.

Similar experiments were performed with pFOXC3-containing mtRNPs. In exogenous reactions having a 98 nucleotide RNA corresponding to the 3' end of the pFOXC3 plasmid having three copies of the 5 bp repeat and using [α-^32^P]dATP with a full complement of nucleotides, labeled products were observed in the range of 20-115 kDa, with the majority of products being below 35 kDa (Figure 3b, lane 1). As was done in experiments using MN-treated pFOXC1 mtRNPs, reactions were performed in the presence and absence of an RNA template and with different combinations of nucleotides. Interestingly, most of these reactions produced a single radiolabeled band that migrated at 60 kDa (Figure 3b, lanes 2-4). The intensity of the band varied depending on the specific combination of unlabeled nucleotides used in the reactions and, unlike reactions with pFOXC1-containing mtRNPs, the generation of this product was not dependent on the presence of an exogenous RNA template. Post-treatment of exogenous reactions with proteinase K eliminated the 60 kDa product and had little effect on the RNA-primed (that is, snapped-back) cDNA products that migrate below 35 kDa (compare lanes 6 and 5). Experiments carried out with mtRNPs isolated from a plasmid-free strain of *F. oxysporum *failed to produce labeled products (data not shown). Collectively, the results suggest that reactions using MN-treated pFOXC3-containing mtRNPs produce a nucleotide-linked protein in the absence of an exogenous RNA template.

### Identification of the nucleotide-linked protein in pFOXC3-containing mtRNPs

**Figure 4:**
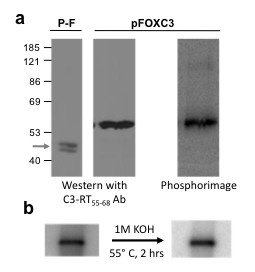
**A nucleotide-linked protein comigrates with pFOXC3-reverse transcriptase (RT) and is unaffected by strong base treatment**. Products of reverse transcription reactions using mitochondrial ribonucleoprotein (mtRNP) particles isolated from a pFOXC3-containing strain or a plasmid-free strain (P-F) having [α-^32^P]dATP and unlabeled deoxyguanosine triphosphate (dGTP) and thymidine triphosphate (TTP) were separated via 10% SDS-PAGE and transferred to nitrocellulose. **(a) **The two panels on the left are from a nitrocellulose membrane probed with protein A-purified pFOXC3-RT_55-68 _rabbit antiserum and visualized by chemiluminesence. The panel on the right is a phosphorimage of radiolabeled protein on the same nitrocellulose membrane. Prestained protein size markers are indicated on the left in kDa. The gray arrow indicates non-specific bands detected in the plasmid-free mtRNP preparation. **(b) **Micrococcal nuclease (MN)-treated pFOXC3-containing mtRNPs were incubated with [α-^32^P]dATP and unlabeled dGTP and TTP. The products were separated via 4-20% gradient SDS-PAGE and radiolabeled products were detected by a phosphorimager. The gel was rehydrated in 1 M KOH and, following incubation at 55°C, the gel was neutralized and dried prior to detection by a phosphorimager.

The predicted size of the pFOXC3-RT polypeptide is 62 kDa, approximately the size of the nucleotide-linked protein detected in reverse transcription reactions using MN-treated pFOXC3-containing mtRNPs. To determine if the labeled protein is the pFOXC3-RT itself, an antibody was generated against a synthetic peptide that corresponds to amino acids 55-68 of the predicted pFOXC3-RT polypeptide (C3-RT_55-68_). This antibody successfully identifies a protein that migrates at 58-60 kDa in pFOXC3-containing mtRNPs and is absent in mtRNPs isolated from a plasmid-free (P-F) strain (Figure 4 and data not shown). The C3-RT_55-68 _antibody was then used to identify the pFOXC3-RT following exogenous reverse transcription reactions. For this experiment, products from reverse transcription reactions using MN-treated pFOXC3-containing mtRNPs were separated by SDS-PAGE and transferred to a nitrocellulose membrane, which was subsequently probed with the C3-RT_55-68 _antibody. A band corresponding to the pFOXC3-RT was identified migrating at 60 kDa (Figure 4a) and this band was absent in mtRNPs from the plasmid-free strain. The membrane was also exposed to phosphorimager analysis and a single radiolabeled band representing the nucleotide-linked protein was detected migrating at the same position as the pFOXC3-RT. These results suggest that the protein covalently linked to DNA in exogenous reverse transcription reactions is the pFOXC3-RT itself.

To provide further evidence that the pFOXC3-RT is the nucleotide-linked protein, we attempted to use the pFOXC3-RT_55-68 _antibody to immunoprecipitate (IP) the labeled protein. Despite making modifications to the buffer, chaotropic agents (both anionic and non-ionic detergents) and antibody affinity resins, no successful combination of antibody-buffer-detergent-bead was found that would selectively capture the nucleotide-linked protein. A separate approach was taken to identify the isolated nucleotide-linked protein via mass spectrometry. Unfortunately, the initial attempt was inconclusive as none of the reported peptides had significant homology to the RT, or to mitochondrial or fungal proteins (data not shown). Thus, at this point, we do not have definitive evidence that the protein labeled in the RT reactions is the pFOXC3-RT, yet it remains the leading candidate and alternative approaches are being explored to identify the labeled protein.

Genetic elements that initiate DNA synthesis via protein priming use a hydroxyl group present in the side chain of a serine, threonine, or tyrosine of the primer to initiate synthesis (reviewed in [13]). Phosphoserine and phosphothreonine linkages have been shown to be labile under strong base treatment, whereas phosphotyrosine linkages are not [20]. To assess which type of amino acid residue is involved in forming the nucleotide linkage, reverse transcription products from MN-treated pFOXC3-containing mtRNPs were separated via SDS-PAGE and incubated under alkaline conditions (1 M KOH, 55°C, 2 h) that were previously shown to liberate phosphoserine and phosphothreonine linkages [20]. Following base treatment, the reaction products retained the ^32^P label, suggesting that the observed linkage occurs through a tyrosine residue of the protein (Figure 4b).

### Nucleotide preference of deoxynucleotidylation

**Figure 5:**
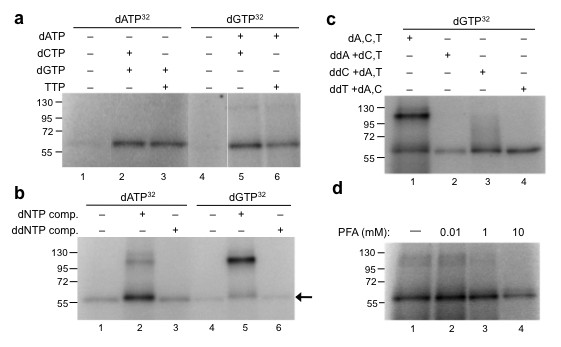
**Analysis of the nucleotide-linked protein in the presence of different combinations of deoxynucleotides and dideoxynucleotides**. Micrococcal nuclease (MN)-treated pFOXC3-containing mitochondrial ribonucleoproteins (mtRNPs) were incubated with [α-^32^P]dATP or [α-^32^P]deoxyguanosine triphosphate (dGTP), and with unlabeled deoxyribonucleotide triphosphates (dNTPs) or dideoxynucleotide triphosphates (ddNTPs), as indicated. Reactions were terminated with Laemmli buffer and reaction products were separated via 4% to 20% SDS-PAGE. Prestained protein size markers are indicated on the left in kDa. **(a) **Reactions having [α-^32^P]dATP or [α-^32^P]dGTP with one unlabeled deoxynucleotide. **(b) **Reactions having [α-^32^P]dATP or [α-^32^P]dGTP, with a full complement of deoxynucleotides or dideoxynucleotides. **(c) **Reactions having [α-^32^P]dGTP and a single dideoxynucleotide with a complement of deoxynucleotides, as indicated. **(d) **Reactions having [α-^32^P]dATP and a full complement of deoxynucleotides incubated with or without phosphonoformate (PFA) at the indicated concentrations. Prestained protein size markers are indicated on the left in kDa and bands showing a slight size difference are indicated by an arrow.

As shown in Figure 3b, the intensity of the 60 kDa nucleotide-linked protein (nucleotide-protein) varied depending on the combination of dNTPs used in the reactions. To investigate the specificity of the deoxynucleotidylation activity that generates the 60 kDa nucleotide-protein product, a comprehensive set of experiments was performed using a radiolabeled dNTP with different combinations of unlabeled dNTPs (summarized in Tables 2 and 3). The 60 kDa nucleotide-protein product was generated using each of the four radiolabeled deoxynucleotides alone; however, when additional nucleotides were included in the reactions, the intensity of the band was markedly higher in reactions having dATP and/or dGTP. In most cases, the addition of one, two or three unlabeled dNTPs resulted in increased levels of the 60 kDa nucleotide-protein product over reactions having a single deoxynucleotide (Table 2). In reactions using [α-^32^P]dATP plus one additional nucleotide, the relative amount of the 60 kDa nucleotide-protein increased slightly with dGTP (1.6-fold) and dCTP (1.3-fold) and was unaffected with TTP, while in reactions having [α-^32^P]dGTP, the addition of dATP led to a threefold increase in band intensity. A more pronounced effect was observed in reactions having two additional unlabeled nucleotides. Figure 5a shows the results of reactions carried out with [α-^32^P]dATP alone and in the presence of dCTP and dGTP or with dGTP and TTP. The intensity of the 60 kDa nucleotide-protein increased more than 10-fold in the presence of two unlabeled dNTPs compared to [α-^32^P]dATP alone (Table 2). This increase in labeled nucleotide-protein product was not as high when dCTP and TTP were used (2.6-fold), and similar results were obtained when using [α-^32^P]dGTP as the label (that is, the highest increase was observed using dATP with dCTP and dATP with TTP, but not dCTP with TTP). Experiments with [α-^32^P]dCTP showed that the addition of dATP with dGTP was the most effective combination (2.4-fold increase). In reactions with [α-^32^P]TTP, all three dinucleotide combinations (that is, dATP with dGTP, dATP with dCTP, and dCTP with dGTP) were effective (8-11-fold increase). While differences were detected depending on the specific nucleotide used as the label, the results of these experiments indicate that the deoxynucleotidylation associated with pFOXC3-containing mtRNPs requires two or more additional deoxynucleotides for maximum labeling and has a strong preference for dATP and dGTP.

**Table 2:** Relative intensity of the 60 kDa nucleotide-linked protein in reactions having different combinations of deoxynucleotides

**Radiolabeled nucleotide**^**a**^	Plus one unlabeled dNTP	Plus two unlabeled dNTPs	Plus three dNTPs
A* (1.0)	G (1.6) > C (1.3) > T (1.0)	CG (17.5) > GT (13.4) > CT (2.6)	CGT (6.3)

G* (1.0)	A (3.0) > C (0.9) > T (0.6)	AC (13.9) > AT (7.9) > CT (1.7)	ACT (4.3)

T* (1.0)	A (1.4) > C (1.3) > G (0.2)	AG (10.8) > AC (8.2) = CG (8.2)	ACG (5.7)

C* (1.0)^b^	A (1.7) > G (1.0) > T (0.6)	AG (2.4) > AT (1.5) > GT (0.7)	AGT (0.7)

^a^Asterisk indicates the radiolabeled nucleotide used in all reactions in the row and numbers in parentheses indicate the intensity of the nucleotide-linked protein product relative to labeling intensity obtained in reactions having a single [α-^32^P]dNTP.

^b^Experiments using [α-^32^P]deoxycytidine triphosphate resulted in non-discrete products that complicated accurate quantification.

dNTP = deoxynucleotide triphosphate.

Experiments using MN-treated pFOXC3-containing mtRNPs with RNAs that correspond to the 3' end of pFOXC3 failed to show the production of a discrete product that represents a protein-linked cDNA like that observed in reactions with pFOXC1-containing mtRNPs. However, when MN-treated pFOXC3 mtRNPs were used in reactions having [α-^32^P]dATP or [α-^32^P]dGTP with a full complement of unlabeled dNTPs in the absence of an RNA, these reactions unexpectedly yielded a radiolabeled cDNA product that migrated at approximately 115 kDa (Figure 3b, lane 1; Figure 5b-d). In a side-to-side comparison in a 10% PAGE gel, this product was found to be smaller than the 120 kDa product observed in reactions having pFOXC1-containing mtRNPs with an exogenous RNA. The 115 kDa product was sensitive to proteinase K treatment and, when analyzed on denaturing 8 M urea polyacrylamide gels, the cDNAs ranged in length with the majority being smaller than 50 nucleotides (not shown). The intensity of the 115 kDa band varied depending on the particular mtRNP preparation, suggesting that it could represent cDNA products that derive from RNA templates protected from micrococcal nuclease treatment. This was further substantiated in experiments using dideoxynucleotide triphosphates (ddNTPs). When ddNTPs were included in place of unlabeled dNTPs, the 115 kDa product was eliminated, whereas the 60 kDa band was unaffected compared to reactions having only the labeled deoxynucleotide (Figure 5b; compare lane 3 with 1, and 6 with 4). In reactions having a single dideoxynucleotide, dideoxy-ATP (ddATP) and dideoxythymidine triphosphate (ddTTP) completely eliminated the 115 kDa product, whereas reactions having dideoxycytidine triphosphate (ddCTP) resulted in products that migrated between 60-115 kDa (Figure 5c). Previous studies of protein-primed reverse transcription of the hepadnaviruses showed that deoxynucleotidylation could be distinguished from DNA synthesis by its insensitivity to the pyrophosphate analog phosphonoformate (PFA; [21]). Figure 5d shows that the formation of the 60 kDa nucleotide-protein product occurs at concentrations of PFA up to 10 mM, whereas the 115 kDa product is substantially reduced. This demonstrates that the 60 kDa and 115 kDa products derive from separate reactions and further suggests that the latter represents a protein-linked cDNA.

The increase in the intensity of the 60 kDa band when using [α-^32^P]dATP or [α-^32^P]dGTP with two or more unlabeled dNTPs suggests that multiple nucleotides are added to a protein in these reactions. A slight increase in the size of the nucleotide-linked protein was also observed in reactions having more than one dNTP (Figure 5b and data not shown). To determine if there was a preferred order of nucleotides, single dideoxynucleotides were included in reactions having a labeled dNTP and two unlabeled dNTPs (Table 3). The inclusion of ddATP inhibited the synthesis of the 60 kDa nucleotide-protein in all cases and resulted in a 40% reduction in band intensity in a reaction using [α-^32^P]dGTP, with dCTP and TTP. The inclusion of dideoxyguanosine triphosphate (ddGTP) inhibited reactions having dATP and dCTP and those with dCTP and TTP, but not with dATP and TTP. These findings suggest that deoxyadenosine monophosphate (dAMP) is added prior to deoxyguanosine monophosphate (dGMP) in the deoxynucleotidylation reactions.

**Table 3:** Relative intensity of the 60 kDa nucleotide-linked protein in reactions having different combinations of deoxynucleotides and dideoxynucleotides

**Radiolabeled nucleotide plus three dNTPs**^**a**^	Plus one ddNTP and two dNTPs				Plus three ddNTPs
		
	ddA	ddG	ddT	ddC	
A*+CGT (1.0)	-	+CT (0.9)	+CG (1.2)	+GT (1.2)	ddCGT (0.2)

G*+ACT (1.0)	+CT (0.6)	-	+AC (1.6)	+AT (1.5)	ddACT (0.3)

T*+ACG (1.0)	+CG (0.4)	+AC (0.6)	-	+AG (0.9)	ddACG (0.3)

C*+AGT (1.0)^b^	+GT (0.5)	+AT (1.2)	+AG (3.3)	-	ddAGT (0.3)

^a^Asterisk indicates the radiolabeled nucleotide used in all reactions in the row and numbers in parentheses indicate the intensity of the 60 kDa nucleotide-linked protein relative to labeling intensity obtained in reactions having a single [α-^32^P]dNTP plus three unlabeled dNTPs.

^b^Experiments using [α-^32^P] deoxycytidine triphosphate resulted in non-discrete products that complicated accurate quantification.

ddNTP = dideoxynucleotide triphosphates; dNTP = deoxynucleotide triphosphate.

### Deoxynucleotidylation precedes cDNA synthesis

**Figure 6:**
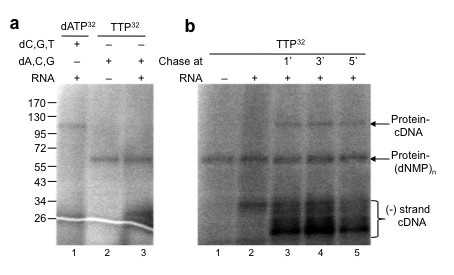
**A nucleotide-linked protein generated with [α-**^**32**^**P]thymidine triphosphate (TTP) in pFOXC1-mitochondrial ribonucleoproteins (mtRNPs) is chased into a protein-cDNA product**. **(a) **Micrococcal nuclease (MN)-treated pFOXC1-containing mtRNPs incubated with [α-^32^P]dATP or [α-^32^P]TTP and a full complement of deoxynucleotides, with or without a 92 nucleotide RNA template, as indicated. **(b) **MN-treated pFOXC1-containing mtRNPs incubated with [α-^32^P]TTP with or without a 92 nucleotide RNA corresponding to the 3' end of pFOXC1. All four unlabeled deoxyribonucleotide triphosphates (dNTPs) were added to reactions to a final concentration of 20 μM at the times indicated. All reactions were terminated with Laemmli buffer and reaction products were separated via 4-20% gradient SDS-PAGE. Prestained protein size markers are indicated on the left in kDa.

The nucleotide preference for the production of the 120 kDa cDNA-protein product associated with pFOXC1-containing mtRNPs was also examined. In reactions with [α-^32^P]dATP and a full complement of unlabeled dNTPs, a 120 kDa protein-linked cDNA was produced. However, in experiments using [α-^32^P]TTP with a full complement of unlabeled dNTPs, an approximately 60 kDa product was detected (Figure 6). This product was generated with and without an exogenous RNA (Figure 6a, lanes 2 and 3), similar to the 60 kDa nucleotide-product observed in reactions with pFOXC3-containing mtRNPs. Since the 3' end of pFOXC1 plasmid RNA is highly A-rich (ending in 4 repeats of CAA), the failure to copy the 92 nucleotide *in vitro *RNA could be impeded without sufficient concentrations of TTP. Increasing the concentration of unlabeled TTP led to a decrease in the intensity of the 60 kDa band due to competition with the radionucleotide (data not shown), yet chasing the reaction with 20 μM dNTPs after 1, 3, and 5 min led to the synthesis of the 120 kDa protein-primed cDNA product (Figure 6b). This finding suggests that deoxynucleotidylation of a 60 kDa protein with TTP precedes the copying of RNA templates and that higher concentrations of TTP are required for elongation.

## Discussion

The evidence presented here suggests that reverse transcription catalyzed by the RTs encoded by the pFOXC mitochondrial retroplasmids is protein primed. The initial observation that the plasmid DNAs have a 5'-linked protein led to speculation that a protein is used to initiate reverse transcription [6] yet protein-linked cDNAs were not observed in previous studies which suggested that other mechanisms of initiation were involved. Prior studies focused on the mechanism of reverse transcription of the pFOXC3 retroplasmid and the efficiency by which the pFOXC3-RT was able to use RNA and DNA primers suggested that nucleic acid primers were employed. In those studies, partially purified pFOXC3-RT preparations were used in reactions having exogenous RNAs that corresponded to the 3' end of the plasmid RNAs, and it was found that cDNA synthesis could initiate by use of complementary DNA oligonucleotides or snapped-back RNAs having minimal base pairing interactions with the template [5]. In this report, we describe unsuccessful attempts to recover RNA:cDNA hybrid molecules among *in vivo *replication intermediates that would represent snapped-back initiation, and when our studies expanded to include the distantly related pFOXC1 retroplasmid, it was found that a high percentage of cDNAs generated from endogenous RNA templates are associated with protein. In experiments using nuclease-treated pFOXC1-containing mtRNPs, a protein-linked cDNA was produced in the presence of an exogenous RNA that corresponds to the 3' end of the plasmid transcript, and in reactions carried out in the absence of an exogenous RNA with a single radiolabeled deoxynucleotide, a 60 kDa nucleotide-linked protein was generated that appears to serve as the primer for reverse transcription.

Heating the products of reverse transcription reactions in the presence of 0.2% SDS prior to separation in SDS-agarose or SDS-polyacrylamide gels made it possible to resolve and analyze protein-linked products. A large portion of (-) strand cDNA products generated from endogenous RNAs associated with pFOXC1-containing mtRNPs was found to be removed by extraction with phenol-CIA, whereas post-treatment of the reactions with proteinase K prevented their removal, indicating that the majority of the labeled cDNAs are associated with protein. When similar reactions were carried out with pFOXC3-containing mtRNPs, a smaller but significant portion of products was also found to be associated with protein. The finding that some of the labeled products were not eliminated by extraction with phenol-CIA suggests that a portion of cDNA products is not attached to protein. This could indicate that nascent protein-primed cDNAs are subject to a cleavage event that removes the protein primer or, since the isolated mtRNPs contain plasmid intermediates at all stages of replication, a fraction of the replication products likely represent pre-existing cDNAs that are extended during the reactions.

Reactions using micrococcal nuclease-treated pFOXC1-containing mtRNPs having an exogenous RNA that corresponds to the terminal 92 nucleotides of the plasmid transcript produced a radiolabeled 120 kDa product when resolved by SDS-PAGE. This product was only observed in reactions having exogenous RNA and was found to be sensitive to post-treatment with proteinase K. The proteinase K-treated reactions contained a new product of approximately 34 kDa which is approximately the size predicted of a full-length cDNA copy of the RNA template, although the precise length of nucleic acids is difficult to determine in these gel systems. The 120 kDa product was also removed by extraction with phenol-CIA and could be recovered from the organic phase of the extraction. Taken together, these results are consistent with those expected of a protein-primed cDNA product. Interestingly, while the 120 kDa product was not detected in reactions that lacked dGTP or TTP, it was synthesized in the absence of dCTP. The reasons for this are unclear, but could reflect the propensity of the pFOXC-RT to misincorporate nucleotides [5].

Reactions carried out with pFOXC3-containing mtRNPs with a similar sized (98 nucleotides) *in vitro *RNA that corresponded to the 3' end of the pFOXC3 plasmid failed to produce a protein-linked cDNA. Instead, a 60 kDa band was produced in the absence of an exogenous RNA. This product was also generated in reaction mixtures having each of the four deoxynucleotides alone or in combination with other dNTPs. Thus, the 60 kDa product appears to represent a deoxynucleotide monophosphate-linked protein (that is, [dNMP]_n_-protein). The 60 kDa product was eliminated by proteinase K treatment and removed by extraction with phenol-CIA (data not shown), confirming its proteinaceous nature. Significantly, the nucleotide-linked protein was found to comigrate with the pFOXC3-RT, suggesting that the RT is self-priming; however, initial efforts to identify the nucleotide-linked protein by immunoprecipitation and mass spectrometry were unsuccessful, thus it remains possible that another protein is involved. Like hepadnaviral RTs, the only other family of RTs known to initiate cDNA synthesis via protein priming, the initiating amino acid appears to be a tyrosine due to the insensitivity of the labeled 60 kDa nucleotide-protein to high alkaline treatment.

Reactions with pFOXC3-containing mtRNPs also generated an approximately 115 kDa product when a full complement of deoxynucleotides was used in the reaction mix. This product appears to represent a protein-primed cDNA as it was found to be sensitive to proteinase K treatment (not shown) as well as to phosphonoformate, which inhibits DNA synthesis without affecting deoxynucleotidylation of proteins that serve as a primer [21,22]. Formation of the 115 kDa product was also strongly suppressed by dideoxynucleotides at concentrations that had little or no effect on the 60 kDa nucleotide-linked protein product. The intensity of the 115 kDa band was also found to vary depending on the MN-treated mtRNP preparation used in the reactions, suggesting that the product could derive from the copying of endogenous RNAs that were incompletely digested or protected during micrococcal nuclease treatment. Similar studies of reverse transcription using MN-treated mtRNPs isolated from strains of *N. crassa *containing the Mauriceville retroplasmid showed that more than 20 nucleotides of plasmid nucleic acid remained associated with the plasmid RT following MN digestion [23].

A comprehensive analysis of the synthesis of the 60 kDa product in reactions with MN-treated pFOXC3-containing mtRNPs under different nucleotide combinations revealed that deoxynucleodtidylation was greatest when three dNTPs were used, and reaction mixtures having dATP and dGTP were the most productive. Reactions having single dideoxynucleotides (together with the appropriate complement of dNTPs) showed that ddATP was the only dideoxynucleotide to inhibit deoxynucleotidylation in all reactions in which it was included, indicating that dAMP is the initial nucleotide incorporated. The next greatest effect was found in reactions that included ddGTP together with [α-^32^P]TTP plus unlabeled dATP and dCTP, in which case the synthesis of the 60 kDa nucleotide-protein was inhibited by more than 40% in this reaction. Taken together, these findings suggest that the first two nucleotides of deoxynucleotidylation are 5'-AG. Since the relative intensity of the nucleotide-labeled product was greatest with three dNTPs, it follows that a third dNTP is necessary for maximum labeling; however, it is unclear from the results whether dCTP or TTP is preferred. The total number of dNMPs incorporated during deoxynucleotidylation also remains to be determined.

When different nucleotide combinations were used in exogenous reactions having MN-treated pFOXC1-containing mtRNPs, it was discovered that a 60 kDa labeled protein was produced in reactions having [α-^32^P]TTP. Like reactions with MN-treated pFOXC3-containing mtRNPs, the generation of this product was not dependent on the addition of an exogenous RNA, indicating that deoxynucleotidylation was not templated by the added RNA; however, based on analysis of the 115 kDa product described above, the priming protein may remain associated with partially digested endogenous RNAs following MN treatment. Interestingly, even in reactions having [α-^32^P]TTP with a full complement of unlabeled dNTPs together with an exogenous RNA, the 120 kDa product was not observed (Figure 6a, lane 3). Yet, when reactions having [α-^32^P]TTP as the sole nucleotide (at a concentration of 0.33 μM) were chased with all four dNTPs (to a final concentration of 20 μM), the 120 kDa band was produced. This suggests that concentrations of TTP greater than 0.33 μM are necessary for the nucleotide-protein complex to engage in cDNA elongation. This finding is consistent with studies of adenoviral protein-primed DNA synthesis that showed that the *K*_m _for elongation was significantly greater than the *K*_m _for initiation [24]. Taken together, these experiments suggest that deoxynucleotidylation occurs prior to reverse transcription.

**Figure 7:**
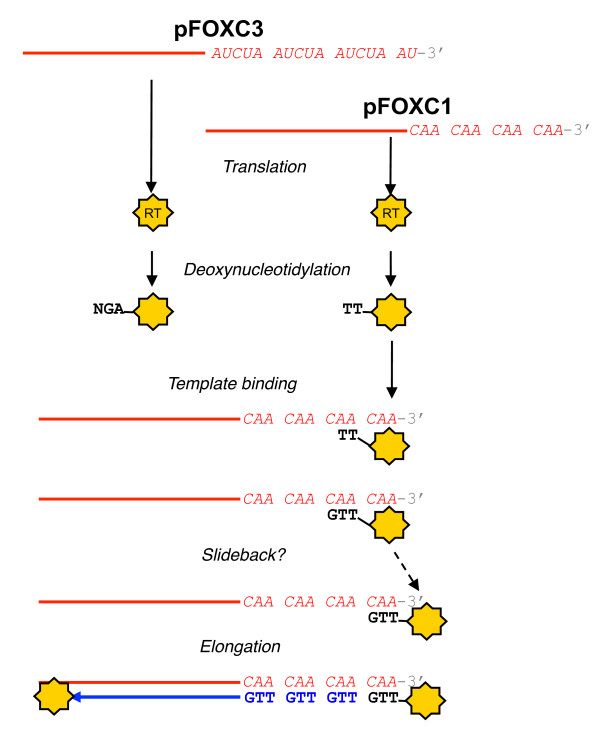
**Model for protein-primed reverse transcription by the pFOXC-reverse transcriptase (RT)**. Transcription of the pFOXC plasmid DNA molecules produces full-length RNAs that appear to function as both mRNAs for the synthesis of the RT and as templates for (-) strand cDNA synthesis [6]. Transcripts of pFOXC3 terminate in approximately three pentameric repeats, whereas transcripts of pFOXC1 terminate in approximately four copies of a 3 bp sequence (the 3' terminus of *in vitro *RNA used in this study is shown). Following production of the plasmid-encoded RT, deoxynucleotidylation occurs with the covalent addition of dAMP to a tyrosine residue of the 60 kDa pFOXC3-RT, followed by incorporation of deoxyguanosine monophosphate (dGMP) and a third nucleotide. Deoxynucleotidylation of the pFOXC1-RT results in the addition of thymidine monophosphate (TMP) to the RT, followed by one or more deoxynucleotide monophosphates (dNMPs) (a second TMP is shown). The resulting RT-(dNMP)_n _complex would have complementarity to the corresponding terminal repeat. Based on studies of protein-primed DNA elements, the model predicts that the complex anneals to the penultimate 3' repeat of the template (shown for pFOXC1 only). Following the synthesis of a unit-length repeat, the RT-(dNMP)_n _complex undergoes a slideback and is repositioned opposite the terminal repeat. The nascent cDNA is elongated via reverse transcription of the template by the 5'-linked RT or by a separate RT recruited to the complex. The model could also accommodate an increase in the number of repeats, depending on the number of slideback events that occur.

The finding that cDNA synthesis catalyzed by the pFOXC-RTs is protein-primed is consistent with earlier models of repeat addition that were based on a slideback mechanism associated with protein-primed elements. In cases where protein priming occurs at the termini of these elements (that is, Ф-29 and adenovirus), the nucleotide that functions as template for the initial deoxynucleotidylation reaction is located 2-4 nucleotides upstream of the 3' terminus. Following the addition of one or more nucleotides, the nucleotide-protein complex undergoes a slideback of 1-3 nucleotides prior to elongation (reviewed in [13]). Collectively, our studies support a model of retroplasmid replication that involves the use of the plasmid RT as primer for reverse transcription and, when combined with the slideback mechanism associated with protein-primed DNA elements, would account for the maintenance and potential extension of the 3' terminal repeats (Figure 7). As previously proposed, the plasmid RNA appears to serve as an mRNA for the synthesis of the RT and as template for (-) strand cDNA synthesis. The initial step of DNA synthesis involves the covalent linkage of dNMP to the protein primer, which is likely to be the pFOXC-RT. *In vitro *experiments indicate that deoxynucleotidylation occurs in the absence of an RNA template (as shown), yet we cannot discount that the RT remains associated with remnants of the endogenous RNA that serve as template for this reaction. Experiments using pFOXC3-containing mtRNPs indicate that deoxynucleotidylation involves the covalent linkage of dAMP to a tyrosine residue of a 60 kDa protein, followed by the incorporation of dGMP and at least one other dNMP. The resulting nucleotide-protein complex would have partial complementarity to the 3' terminal repeat (5'-AUCUA) of the plasmid transcript. Likewise, in reactions using pFOXC1-containing mtRNPs, deoxynucleotidylation leads to the incorporation of one or more thymidine monophosphate (TMP) molecules attached to a 60 kDa protein and the resulting [TMP]_n_-protein complex would have complementarity to the 3' terminal repeat (5'-CAA) of the pFOXC1 plasmid transcript. Based on analogies to protein-primed linear DNAs, the TMP-labeled protein associates with the A residues of the penultimate repeat and one or more nucleotides are added prior to a slideback that would reposition the nascent cDNA opposite the terminal repeat. It is not known if a second RT molecule is involved in elongation (as shown) or whether the RNA is spooled through the active site of the attached RT. The resulting (-) strand cDNA would maintain the integrity of the terminal repeats and would be protected by a 5'-linked protein. The model could also accommodate more than one slideback, as has been demonstrated with DNA slidebacks associated with the PRD1 bacteriophage [13]. This would potentially extend the length of the repeated region and ensure that sequence information is not lost during replication.

The finding that the pFOXC plasmids replicate via protein-primed reverse transcription makes them only the second retroelement family known to use a protein to initiate cDNA synthesis. Amino acid sequence comparisons fail to identify regions of the pFOXC-RTs that show high similarity to the terminal protein domain of hepadnaviral RTs. The pFOXC-RTs also lack homology to the TPs of protein-primed DNA elements or genome-linked proteins of RNA viruses (VPgs). Yet, despite the surprisingly high degree of evolutionary divergence between the pFOXC1-RTs and pFOXC2/pFOXC3-RTs, it is noteworthy that a 20 amino acid region of high similarity (greater similarity than that of the conserved RT domains) is detected near the amino terminus and contains a tyrosine residue that could potentially serve as the primer. Efforts are underway to express the RTs in heterologous hosts to demonstrate the importance of this region and further characterize the mechanism of cDNA initiation.

## Conclusions

We provide evidence that the pFOXC retroplasmids initiate reverse transcription by use of a protein primer, making them only the second retroelement lineage known to use a protein to prime cDNA synthesis. When combined with previous studies, the pFOXC-RTs appear to be unique among polymerases in their ability to use RNA, DNA or protein to initiate DNA synthesis. This provides additional support of the hypothesis that mitochondrial retroelements represent a type of 'molecular fossil' that hold clues about the evolutionary past. Our findings also suggest that the role of protein-primed replication in maintaining the termini of linear genetic elements is not restricted to elements that replicate using DNA-dependent DNA polymerases or RNA-dependent RNA polymerases, and can include elements that replicate via reverse transcription. Whether this is a case of convergent evolution or represents a primordial feature conserved among polymerases remains to be determined. It is intriguing that the 3' terminal repeats of the pFOXC1 and pFOXC2/pFOXC3 retroplasmid families have such marked differences, both in length and sequence. If, as proposed, a slideback mechanism is involved in the maintenance of the terminal repeats, further study of the pFOXC retroplasmids could reveal mechanistic adaptations of the RT to accommodate the progressive lengthening of slidebacks. These studies could also provide insight into the mechanistic ability of the RTs associated with telomerases (TERTs) to add short DNA repeats via iterative slidebacks on an RNA template, as well as the duplication of 3' repeats during retrotransposition of certain non-long terminal repeat (non-LTR) retroelements.

## Methods

### *F. oxysporum *strains and growth conditions

Strains used in this study were *F. oxysporum *777, f. sp. *conglutinans *(pFOXC1-containing strain), *F. oxysporum *725, f. sp. *matthioli *(pFOXC3-containing strain) and plasmid-free strain *F. oxysporum *9129, f. sp. *cubense*. These strains are maintained at the US Department of Agriculture Agricultural Research Service (USDA-ARS) Cereal Disease Laboratory (St Paul, MN, USA). Strains were grown on potato dextrose agar plates, and conidia preserved in 20% glycerol and stored at -80°C. Conidia were germinated for 3-4 days in 1-2 l of 1 × Vogel's medium [25] shaking at 150 rpm at 25°C.

### Isolation of mitochondria and mtRNP particles

Mitochondria were prepared from mycelia by the flotation gradient method [26]. Mitochondrial RNP complexes were isolated by resuspending mitochondrial pellets in 3.5 ml of 25 mM Tris, pH 7.5, 500 mM KCl, 25 mM CaCl_2_, 20 mM dithiothreitol (HKCT-D) and lysed by addition of Nonidet P-40 to a final concentration of 1%. Lysates were layered over a 1.85 M sucrose cushion containing HKCT-D, and centrifuged in a Beckman Ti50 rotor (226,000 *g*, 17 h, 4°C; [27]). Mitochondrial RNP pellets were stored at -80°C and resuspended in a solution of 50 mM Tris-HCl, pH 8.2, 0.5 mM ethylenediaminetetra-acetic acid (EDTA), 10 mM KCl, and 5 mM dithiothreitol (DTT) at concentrations of 1-2 A_260 _OD units/μl.

### Cloning of the termini of pFOXC1 and (-) strand cDNA products

Plasmid pFOXC1 was isolated from agarose gels and subjected to digestion with exonuclease III (New England Biolabs, Beverly, MA, USA) to cleave single-stranded regions in the hairpin or used directly in tailing reactions using terminal deoxynucleotide transferase (Promega, Madison, WI, USA) and dGTP. The tailed products were amplified by anchored PCR using plasmid-specific primers C1 5' (5'-GCTGGATCCCCGACACTGATTCATG) or C1 3' (5'-GAAGGATCCAGTATCAAATGGGGACTC), and dCBAM (ATATAGGAC_16_). Products were digested with *Bgl*II and *Bam*HI, cloned into the *Bam*HI site of Bluescribe (pBS; Stratagene, La Jolla, CA, USA) and sequenced.

### Reverse transcription reactions

Mitochondrial RNP particles were used directly in reactions (that is, endogenous reactions) or were treated with nuclease to degrade nucleic acids prior to use in reactions having added RNAs (that is, exogenous reactions). Endogenous reactions included 0.4 A_260 _OD units of mtRNP particles in a solution having 50 mM Tris-HCl, pH 8.2, 5 mM MgCl_2_, 50 mM KCl, 20 μCi of [α-^32^P]dCTP, 125 μM dATP, dGTP and TTP and 5 mM DTT. Unless otherwise indicated, mtRNP particles were preincubated with actinomycin D (100 μg/ml, Sigma-Aldrich, St Louis, MO, USA) for 5 min at 4°C prior to addition to reaction mixtures. Reactions were incubated at 37°C for 15 min, then chased by the addition of dCTP to 100 μM and incubated for an additional 10 min. Reactions were stopped by the addition of EDTA to a concentration of 125 mM prior to post-treatment and/or precipitation with ethanol. Post-treatment of endogenous reactions included incubation with proteinase K at 50°C for 15 min with and without extraction with equal volume phenol-CIA (25:24:1). Recovery of products from phenol was carried out by precipitation with four volumes of 100% acetone. Precipitated products were resuspended in 10 mM Tris-HCl, pH 7.0, 1 mM EDTA (TE) and quantified as previously described [6,28]. Endogenous products were directly separated on 1.0% to 1.2% agarose gels, or were incubated in a loading dye mixture containing 0.2% SDS and heated for 5 min at 65°C prior to loading on a 1.0% to 1.2% agarose gel containing 0.2% SDS. For exogenous reactions, the pFOXC-RT was released from the mtRNP particles by degrading endogenous nucleic acids with MN (Takara Bio USA, Madison, WI, USA). In most cases, 5-10 A_260 _OD units were incubated in a reaction having 50 mM Tris, pH 8.2, 1 mM CaCl_2 _with 5 IU MN/A_260 _OD unit for 15 s at 37°C followed by 10 min at room temperature. To inhibit the MN activity, EGTA was added to a concentration of 10-20 mM. The MN-treated mtRNPs were incubated at 4°C with 100 μg/ml actinomycin D prior to use in reaction mixtures having 1-2 μg of an *in vitro *synthesized RNA template. Reverse transcription reactions were carried out in a reaction buffer having 50 mM Tris-HCl, pH 8.2, 20 mM MgCl_2_, and 0.33 μM [α-^32^P]dNTP and/or 20 μM dNTPs and/or 100 μM ddNTPs, as indicated. Where indicated, reactions were post-treated with proteinase K (0.2 mg/ml) at 50°C for 15 min, with or without extraction with equal volume of phenol-CIA. Reactions were boiled in Laemmli buffer (125 mM Tris-HCl, 2% SDS, 10% glycerol, 5% 2-mercaptoethanol) for 5 min, and separated via 7.5%, 10% or 4-20% gradient SDS-PAGE, as indicated. Gels were electrophoresed for 1-2 h at 120 volts and then fixed in a solution of 20% ethanol and 10% glycerol for 30-45 min, and dried in a drying apparatus. Dried gels were exposed to phosphorimager screen overnight and analyzed by a Storm 860 Phosphorimager and ImageQuant V.5.2 (GE Healthcare Biosystems, Piscataway, NJ, USA). Where indicated, products in gels were transferred to a nitrocellulose membrane for detection by phosphorimager and western blot analysis.

### Synthesis of *in vitro *RNA templates

Templates for RNA synthesis of the pFOXC3 transcript containing three repeats were generated and transcribed as previously described [5]. Templates for the synthesis of the 92 nucleotide pFOXC1 RNA were generated by amplification of a clone containing the 3' terminal region of pFOXC1 with a primer having a T7 promoter sequence (C192nt+*Bam*; 5'-CCGGATCCTAATACGACTCACTATAGGCTGAGGAAATTTG) and another corresponding to the end of the plasmid having 4 copies of the repeat (C14R+ *Eco*; 5'-CCGAATTCTTGTTGTTGTTGTTTCCAACCTC). This fragment was inserted into the multiple cloning site of pBluescribe. An amplicon was generated using a pBS forward primer and C1 92nt 4R (5'-TTGTTGTTGTTGTTTCCAACCTC) and used as a template to generate run off transcripts. *In vitro *transcription was carried out in a 20 μl reaction volume containing 100 ng of template DNA, reaction buffer containing 40 mM Tris-HCl, pH 7.9, 6 mM MgCl_2_, 10 mM DTT, 2 mM spermidine, and 50 IU of T7 RNA polymerase (New England Biolabs, Ipswich, MA, USA). Reactions were incubated 30-60 min at 37°C. DNA was digested with 2 IU RQ1 DNase (Thermo Fisher Scientific, Waltham, MA, USA) in 40 mM Tris-HCl, pH 8.0, 10 mM MgSO_4_, 1 mM CaCl_2_, for 30 min at 37°C. Transcripts were extracted with phenol-CIA, precipitated with ethanol, and resuspended in dH_2_O.

### Development of an antibody to pFOXC3-RT and western blot analyses

A synthetic peptide derived from the predicted pFOXC3 polypeptide corresponding to positions 55-68 (KEVKRANRYLAFQE) was synthesized and used in the production of a rabbit polyclonal antibody (Sigma Genosys, Woodlands, TX, USA). Following electrophoresis, protein samples were electroblotted to nitrocellulose (0.45 μm; MSI Laboratories, Westboro, MA, USA) in transfer buffer (25 mM Tris, pH 8.5, 200 mM glycine, 20% methanol). The membrane was washed in Tris-buffered saline (TBS; 20 mM Tris, pH 7.6, 125 mM NaCl) with 0.1% Tween 20 and incubated for 1 h in blocking buffer (5% non-fat dry milk, 0.1% Tween 20 in TBS). The membrane was washed again and incubated with protein-A purified pFOXC3-RT_55-68 _antibody in blocking buffer for 2-16 h. Following incubation with primary antibody, the membrane was washed and incubated with horseradish peroxidase (HRP)-linked anti-rabbit IgG secondary antibody (1:2,000; Cell Signaling Technology, Beverly, MA, USA) in blocking buffer for 1 h. The membrane was washed and incubated with a chemiluminescent HRP substrate (Thermo Scientific, Rockford, IL, USA) for 5 min, followed by detection using x-ray film and analysis with a LAS-4000 chemiluminescent imaging reader (Fujifilm, Tokyo, Japan).

### Analysis of nucleotide-amino acid linkage

Exogenous reverse transcription products were separated via 4-20% gradient SDS-PAGE, and the gel was dried and subjected to phosphorimager analysis. The gel was then rehydrated in 10 gel volumes of 1 M KOH and incubated at 55°C for 2 h, then neutralized with four changes of 10% acetic acid/10% isopropanol. The gel was dried again and exposed to phosphorimager analysis.

The sequence of the pFOXC1 plasmid was deposited in GenBank under the accession ID number HQ026775.

## Competing interests

The authors declare that they have no competing interests.

## Authors' contributions

JG was responsible for the execution and analysis of exogenous reverse transcription assays and western blots, participated in experimental design, construction of figures for the manuscript, drafting and editing of the manuscript. SM was responsible for execution and analysis of endogenous and exogenous reverse transcription assays and editing of the manuscript. JK conceived of the study, was responsible for its design and coordination, and drafted the manuscript. All authors read and approved of the final manuscript.

## Supplementary Material

Additional file 1 **Supplementary Table 1 **Sequence of clones of the terminus of pFOXC1. Click here for file

Additional file 2 **Supplementary Table 2 **Reverse transcriptase activity associated with mitochondrial ribonucleoproteins (mtRNPs) containing pFOXC1 or pFOXC3. Click here for file
