# Supplementary material for: Reverse transcription of the pFOXC mitochondrial retroplasmids of Fusarium oxysporum is protein primed
Source: Mob DNA. 2011 Jan 21;2:1. doi: 10.1186/1759-8753-2-1 (PMC3035579; doi:10.1186/1759-8753-2-1)
Supplement: Additional file 1 — Supplementary Table 1 Sequence of clones of the terminus of pFOXC1. [file 1759-8753-2-1-S1.DOCX]

### Supplementary Table 1: Sequence of clones of the terminus of pFOXC1

| 3’ DNA sequence | Number of Clones |
| --- | --- |
| GAAGAAAAAAACTCTAAGAGGTTGGAAA CAA CAA CAA-3’ | 2 |
| “AAGAAAAAAACTCTAAGAGGTTGGAA “ CAA CAA CAA C | 4 |
| “AAGAAAAAAACTCTAAGAGGTTGGAA “ CAA CAA CAA CA | 3 |
| “AAGAAAAAAACTCTAAGAGGTTGGAA “ CAA CAA CAA CAA | 3 |
| “AAGAAAAAAACTCTAAGAGGTTGGAA “ CAA CAA CAA CAA C | 1 |
