# Supplementary material for: Reverse transcription of the pFOXC mitochondrial retroplasmids of Fusarium oxysporum is protein primed
Source: Mob DNA. 2011 Jan 21;2:1. doi: 10.1186/1759-8753-2-1 (PMC3035579; doi:10.1186/1759-8753-2-1)
Supplement: Additional file 2 — Supplementary Table 2 Reverse transcriptase activity associated with mitochondrial ribonucleoproteins (mtRNPs) containing pFOXC1 or pFOXC3. [file 1759-8753-2-1-S2.DOCX]

**Supplementary Table 2: Reverse transcriptase activity associated with pFOXC1- and pFOXC3-containing mtRNPs**

| Strain | Standard Conditions^1^ | +Actinomycin D^2^ | +RNase^3^ |
| --- | --- | --- | --- |
| 777 (pFOXC1) | 19,930 ± 360 (100) | 13,300 ± 560 (67) | 630 ± 50 (3.1) |
| 725 (pFOXC3)^4^ | 50,800 ± 790 (100) | 55,300 ± 4,430 (109) | 920 ± 60 (1.8) |
| 9129 (plasmid-free)^4^ | 630 ± 150 | 40 ± 10 | – |

^1^ Reactions included 0.04 O.D. units (approximately 5 ng of protein) of mtRNP particles in a mixture having 50 mM Tris-HCl, 5 mM MgCl_2_, 50 mM KCl, 5 mM DTT, 100 µg/ml, 125 µM dATP, dGTP, TTP, and 20 µCi of [α-^32^P]dCTP. Reactions were incubated at 42˚ C for 15 min, then chased by the addition of dCTP to 100 μM and incubated for an additional 10 min at 42˚ C. Reactions were stopped by the addition of 20 ul of 0.25 M EDTA and precipitated. Steps taken to remove the unincorporated nucleotides and quantification of the amount of [α-^32^P]dCTP incorporated into high molecular products (measured by CPM) are described in 6. Numbers in parentheses indicate the percent of the activity detected under standard conditions.

^2^ Reactions were preincubated with actinomycin D (100 μg/ml) for 5 min at 4˚ C, prior to the addition of the labeled nucleotide.

^3^ Reactions were preincubated with 0.1 U of RNase A for 5 min at 22˚ C, prior to the addition of the labeled nucleotide.

^4^ Previously reported in 6, yet carried out with the reactions having pFOXC1 mtRNPs
